# The role of cGAS in epithelial dysregulation in inflammatory bowel disease and gastrointestinal malignancies

**DOI:** 10.3389/fphar.2024.1409683

**Published:** 2024-07-10

**Authors:** Anna Ramos, Nazih Bizri, Elizabeth Novak, Kevin Mollen, Sidrah Khan

**Affiliations:** ^1^ Department of Surgery, University of Pittsburgh Medical Center, Pittsburgh, PA, United States; ^2^ Division of Pediatric General and Thoracic Surgery, UPMC Children’s Hospital of Pittsburgh, University of Pittsburgh Medical Center, Pittsburgh, PA, United States

**Keywords:** cGAS, STING, intestinal epithelium, inflammatory bowel disease, gastrointestinal malignancy

## Abstract

The gastrointestinal tract is lined by an epithelial monolayer responsible for selective permeability and absorption, as well as protection against harmful luminal contents. Recognition of foreign or aberrant DNA within these epithelial cells is, in part, regulated by pattern recognition receptors such as cyclic GMP-AMP synthase (cGAS). cGAS binds double-stranded DNA from exogenous and endogenous sources, resulting in the activation of stimulator of interferon genes (STING) and a type 1 interferon response. cGAS is also implicated in non-canonical pathways involving the suppression of DNA repair and the upregulation of autophagy via interactions with PARP1 and Beclin-1, respectively. The importance of cGAS activation in the development and progression of inflammatory bowel disease and gastrointestinal cancers has been and continues to be explored. This review delves into the intricacies of the complex role of cGAS in intestinal epithelial inflammation and gastrointestinal malignancies, as well as recent therapeutic advances targeting cGAS pathways.

## Introduction

Gastrointestinal (GI) epithelial cells serve as the first line of defense against harmful luminal contents. These cells have a host of defense mechanisms, including selective permeability, production of antimicrobial peptides, and immune surveillance (Ramanan and Cadwell, 2016). An intricate relationship exists between this barrier and the immune system responsible for the second and third lines of defense (Wittkopf et al., 2014). Maintaining a constant balance between these lines of defense is pivotal for homeostasis, protection against pathogenic threats, and prevention of autoimmunity (Wittkopf et al., 2014; Okumura and Takeda, 2017). A key player in this dynamic relationship is cyclic GMP-AMP synthase (cGAS).

cGAS is a pattern recognition receptor that detects and binds to cytoplasmic DNA (Shu et al., 2014). Upon binding double-stranded DNA (dsDNA) from either exogenous or endogenous sources, cGAS activates the stimulator of interferon genes (STING), leading to downstream production of type 1 interferons and other inflammatory mediators (Cheng et al., 2020). Recent studies have demonstrated that cGAS not only activates the innate immune response but also influences DNA repair mechanisms and autophagy, highlighting its critical role beyond that of merely sensing cytosolic DNA (Liang et al., 2014; Liu et al., 2018; Cheng et al., 2020). A growing body of research demonstrates the importance of abnormal cGAS signaling in the pathogenesis of various GI diseases. In ulcerative colitis, abnormal activation of cGAS contributes to the dysregulation of intestinal epithelial autophagy, epithelial cell integrity, and innate immune responses (Ke et al., 2022; Yang et al., 2023). Meanwhile, in GI cancers, cGAS-mediated pathways have been shown to be both oncogenic and tumor suppressive (Ke et al., 2022; Yang et al., 2023). As such, understanding the nuanced role of cGAS in these conditions offers promising avenues for therapeutic intervention.

In this comprehensive review, we explore the various cGAS signaling pathways, the role of cGAS dysregulation in inflammatory bowel disease (IBD) and GI malignancies, and the therapeutic interventions targeting these pathways. Through this exploration, this review aims to shed light on the complex role of cGAS in GI health and disease, paving the way for future research and therapeutic strategies.

## cGAS signaling pathways

### Structure, localization, and activation of cGAS

**FIGURE 1:**
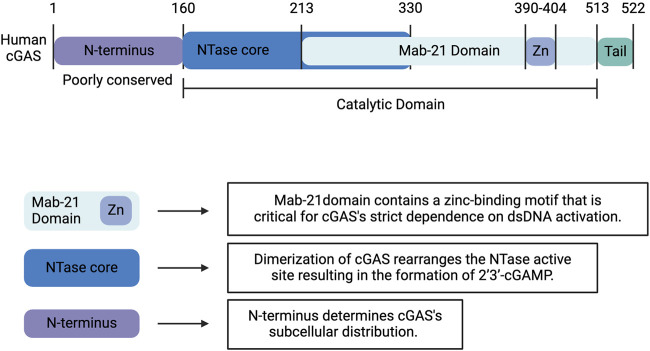
Structure of Human cGAS. Human cGAS is a 522-amino acid, DNA-sensing nucleotidyltransferase (NTase) composed of a highly conserved C-terminal domain and an unstructured, poorly conserved N-terminal domain. The C-terminal fragment or catalytic domain contains an NTase core scaffold appended to a zinc-binding motif. This zinc-ribbon, DNA-binding domain is critical for cGAS’s strict dependence on B-form dsDNA activation. Upon binding dsDNA, cGAS dimerizes, which produces a conformational change that rearranges the NTase active site and results in the formation of 2′3′-cyclic GMP-AMP (2′3′-cGAMP). Whereas the C-terminal domain influences the catalytic activity of cGAS, the less well-characterized and poorly conserved N-terminus determines the subcellular distribution. Created with BioRender.com.

**FIGURE 2:**
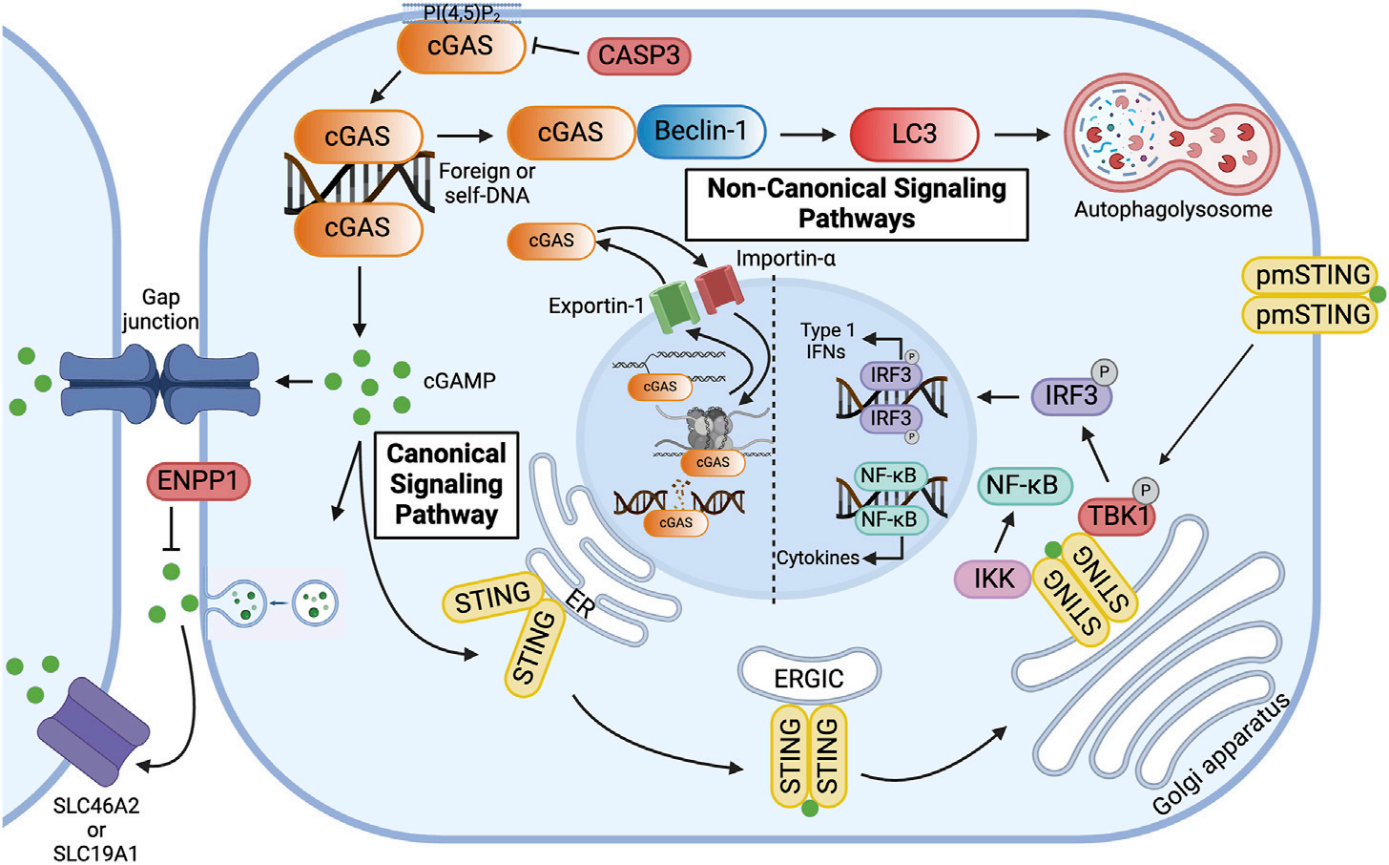
Overview of Canonical and Non-canonical cGAS Signaling Pathways. cGAS is primarily localized to the plasma membrane via binding of the N-terminus to phosphatidylinositol 4, 5-bisphosphate [PI(4, 5)P_2_]. In the canonical signaling pathway, cyclic GMP-AMP synthase (cGAS) binds double-stranded DNA (dsDNA) in the cytoplasm, resulting in the formation of 2′3′-cyclic GMP-AMP (cGAMP). cGAMP activates the endoplasmic reticulum (ER) membrane protein stimulator of interferon genes (STING), which dimerizes and travels to the Golgi apparatus via the ER-Golgi intermediate compartment (ERGIC). At the Golgi apparatus, STING recruits Tank-binding kinase 1 (TBK1) and interferon regulatory factor 3 (IRF3) and activates IKB kinase (IKK), thereby inducing the expression of type 1 interferons and pro-inflammatory cytokines. To avoid overactivation and maintain immune homeostasis, human cGAS is cleaved by caspase-3 (CASP3). Alternatively, cGAMP can be transferred to neighboring cells via gap junctions or be packaged in viral particles or extracellular vesicles for distant transmission. In the extracellular space, cGAMP can bind to plasma membrane-localized STING (pmSTING) on neighboring cells, imported into the cytosol of bystander cells via plasma membrane proteins, such as SLC46A2 or SLC19A1, or, alternatively, be hydrolyzed by ecto-nucleotide pyrophosphatase/phosphodiesterase (ENPP1). In the non-canonical signaling pathway, cGAS, activated by dsDNA, binds to Beclin-1, which allows for lipidation of microtubule-associated light chain 3 (LC3) and results in autophagy-mediated degradation of pathogenic DNA. cGAS can be transported in and out of the nucleus via Importin-α and Exportin-1, respectively. In the nucleus, cGAS preferentially binds to nucleosomal DNA. When activated, however, cGAS can inhibit homologous recombination of double-stranded DNA breaks, thereby promoting tumorigenesis, or it can alternatively bind to DNA, decelerating replication forks and suppressing replication-induced DNA damage. Created with BioRender.com.

Human cGAS is a 522-amino acid, DNA-sensing nucleotidyltransferase (NTase) composed of a highly conserved C-terminal domain and an unstructured, poorly conserved *N*-terminal domain (Figure 1) (Kranzusch et al., 2013). The C-terminal fragment or catalytic domain contains an NTase core scaffold appended to a zinc-binding motif. This zinc-ribbon, DNA-binding domain is critical for cGAS’s strict dependence on B-form dsDNA activation (Figure 1) (Civril et al., 2013; Kato et al., 2013; Kranzusch et al., 2013). cGAS is activated by dsDNA in a sequence-independent but length-dependent manner (Civril et al., 2013; Andreeva et al., 2017; Luecke et al., 2017; Du and Chen, 2018; Zhou et al., 2018). Upon binding dsDNA, cGAS dimerizes, thereby sandwiching two strands of dsDNA between two cGAS monomers (Shu et al., 2014; Bai and Liu, 2022). This dimerization produces a conformational change that rearranges the NTase active site and results in the formation of a cyclic GMP-AMP dinucleotide comprised of a 2′-5′ and a 3′-5′ phosphodiester linkage (2′3′-cGAMP), which functions as an endogenous second messenger (Figure 2) (Ablasser et al., 2013a; Gao et al., 2013; Zhang et al., 2014). Interestingly, human and mouse cGAS share <60% amino acid identity. The human specific adaptations in NTase and DNA-binding domains have been shown to enhance the specificity of human cGAS for long-stranded DNA and restrain the production of 2′3′-cGAMP, thereby reducing the risk of sterile inflammation (Zhou et al., 2018; Bai and Liu, 2022).

Whereas the C-terminal domain has been shown to influence the catalytic activity of cGAS, the less well-characterized and poorly conserved N-terminus has been implicated in its subcellular distribution (Figure 1) (Shu et al., 2014; Bai and Liu, 2022). cGAS is notably located in both the nucleus and cytoplasm. In the nucleus, cGAS preferentially binds to the acidic patch of the histone H2A-H2B dimer and nucleosomal DNA, which inhibits cGAS dimerization and activation (Figure 2) (Boyer et al., 2020; Kujirai et al., 2020; Michalski et al., 2020; Pathare et al., 2020; Zhao et al., 2020; Bai and Liu, 2022). In fact, nuclear cGAS is estimated to be at least 200-fold less active toward endogenous nuclear DNA than exogenous DNA (Gentili et al., 2019). Nuclear viral DNA, however, can trigger release of cGAS from the nucleosome, resulting in the production of 2′3′-cGAMP (Wu et al., 2022). Translocation from the nucleus to the cytoplasm is directed by the functional nuclear export signal, ^169^ LEKLKL^174^, and mediated by the exportin, chromosomal region maintenance 1 (Figure 2) (Sun et al., 2021). Similarly, cGAS contains two nuclear localization sequences, NLS1 and NLS2. The latter is required for the importin-α-dependent translocation of cGAS from the cytoplasm to the nucleus (Figure 2). In the cytoplasm, cGAS is primarily localized to the plasma membrane via binding of the N-terminus to phosphatidylinositol 4,5-bisphosphate [PI(4,5)P_2_] (Figure 2) (Barnett et al., 2019). This localization is thought to allow for detection of pathogenic DNA while preventing excessive recognition of self-DNA. To avoid overactivation and maintain immune homeostasis, human cGAS is cleaved by caspase-3 (Figure 2) (Ning et al., 2019). Furthermore, during mitosis, barrier-to-autointegration factor 1 (BAF) competes for DNA binding, and phosphorylation of human cGAS at S305 by the major mitotic kinase CDK1-cyclin B complex inhibits the synthesis of 2′3′-cGAMP (Guey et al., 2020; Zikhong et al., 2020; Li et al., 2021a).

### Canonical cGAS signaling pathway

2′3′-cGAMP is an endogenous second messenger synthesized by cGAS that can function as an immunotransmitter. This second messenger can be transferred to neighboring cells via gap junctions or be packaged in viral particles or extracellular vesicles for distant transmission (Figure 2) (Ablasser et al., 2013b; Gentili et al., 2015; Chen et al., 2016a; Zhou et al., 2020; Maltbaek et al., 2022). This transfer mechanism allows for signaling to be passed through local tissue. In particular, this is important for immune and epithelial cells in orchestrating immune responses, although this transfer is not limited to solely these cell types. In the extracellular space, 2′3′-cGAMP is imported into the cytosol of bystander cells via plasma membrane proteins, such as SLC46A2 or SLC19A1 or, alternatively, hydrolyzed by ecto-nucleotide pyrophosphatase/phosphodiesterase (ENPP1), which thereby negatively regulates the cGAS-STING pathway (Figure 2) (Li et al., 2014; Kato et al., 2018; Ritchie et al., 2019; Carozza et al., 2020; Cordova et al., 2021).

Intracellularly, 2′3′-cGAMP activates the innate immune adaptor STING (Figure 2) (Sun et al., 2021; Wu et al., 2022). STING is an endoplasmic reticulum (ER) membrane protein that contains an *N*-terminus with four transmembrane segments and a C-terminus with a cytoplasmic ligand-binding and signaling domain (Shang et al., 2012; Shang et al., 2019). At the ER, calcium sensor protein, stromal interaction molecule 1 (STIM1), binds to the N-terminal transmembrane domains of STING monomers, thereby retaining STING to the ER membrane and preventing spontaneous activation (Srikanth et al., 2019). Upon binding intracellular 2′3′-cGAMP, however, the cytoplasmic domains, which form a dimer, undergo a conformational change (Figure 2) (Shang et al., 2012; Ergun et al., 2019; Shang et al., 2019; Ergun and Li, 2020). This “closing” of the STING homodimer disrupts the interaction between STING and STIM1 and promotes the interaction between STING and CxORF56, also known as STING ER exit protein (STEEP) (Cheng et al., 2020). STEEP recruits GTPase Sar1 and PI(3)K VPS34 complex one to the ER, which initiates COPII-mediated export to the Golgi apparatus through the ER-Golgi intermediate compartment (ERGIC) (Cheng et al., 2020; Zhang et al., 2020; Zheng, 2020). To sense extracellular 2′3′-cGAMP, however, an alternatively spliced STING isoform that lacks one transmembrane domain localizes to the plasma membrane. The C-terminal tail of plasma membrane-localized STING (pmSTING) binds to 2′3′-cGAMP outside the cell, dimerizes, and translocates to the perinuclear area (Figure 2) (Li et al., 2022a).

At the Golgi apparatus, STING undergoes palmitoylation and binds to tank-binding kinase 1 (TBK1) via a conserved PLPLRT/SD motif within the C-terminal tail (Figure 2) (Mukai et al., 2016; Ogawa et al., 2018; Zhang et al., 2019; Zhao et al., 2019; Ergun and Li, 2020). TBK1 in turn phosphorylates STING at S366 in the pLxIS motif, which serves as a docking site for the transcription factor interferon regulatory factor 3 (IRF3) (Figure 2) (Tao et al., 2016; Cheng et al., 2020; Dalskov et al., 2020). Polymerized STING is hypothesized to act as a scaffold for TBK1 and IRF3 (Ergun and Li, 2020). Upon phosphorylation of IRF3 by TBK1, IRF3 translocates into the nucleus and induces the expression of type 1 interferons (IFNs) (Figure 2) (Ishikawa et al., 2009; Abe et al., 2013; Liu et al., 2015; Zheng, 2020). Type 1 IFNs elicit antiviral and immunomodulatory responses and initiate cell-mediated immunity (Schneider et al., 2014). The cGAS-STING pathway also activates nuclear factor kappa B (NF-κB), which induces the expression of pro-inflammatory cytokines, including interleukin-1 (IL-1), interleukin-6 (IL-6), and tumor necrosis factor-α (TNF-α) (Figure 2) (Abe and Barber, 2014; Balka et al., 2020; Cheng et al., 2020). To attenuate the signaling pathway, post-Golgi STING vesicles are degraded by Rab7-positive endolysosomes (Gonugunta et al., 2017). Activation of NF-κB inhibits the trafficking of STING to lysosomes by inducing microtubule depolymerization, thereby enhancing STING signaling (Zhang et al., 2023). Interestingly, in addition to the canonical immunomodulatory pathway, STING has also been shown to activate autophagy at the ERGIC in response to 2′3′-cGAMP by inducing lipidation of microtubule-associated protein 1 A/1B-light chain 3 (LC3) through a mechanism independent of TBK1 and IRF3 activation (Gui et al., 2019; Cheng et al., 2020; Taguchi et al., 2021).

### Non-canonical cGAS signaling pathways

cGAS has been implicated in various STING-independent signaling pathways. In the cytoplasm, for instance, cGAS has been shown to induce macroautophagy. In the presence of dsDNA, the NTase domain of cGAS binds to the autophagy protein, Beclin-1, thereby displacing Rubicon, a negative autophagy regulator, and activating phosphatidylinositol 3-kinase class III (PI3KC3) (Figure 2) (Liang et al., 2014). This interaction not only halts the production of 2′3′-cGAMP due to the inactivation of the NTase domain but also stimulates autophagy-mediated degradation of pathogenic DNA. Similarly, cGAS mediates the autophagy of micronuclei, a hallmark of genome instability and trigger of innate immunity, by interacting directly with an essential autophagy protein, LC3 (Zhao et al., 2021). Both mechanisms prevent excessive cGAS activation and dampen innate immune surveillance.

In the nucleus, however, cGAS has been shown to *stimulate* immune surveillance by regulating the histone arginine modification at the *Ifnb* and *Ifna4* promoters, thereby facilitating chromatin accessibility and production of type 1 IFNs (Cui et al., 2020). Additionally, nuclear cGAS can both promote and oppose genomic instability. DNA damage induces importin-α-dependent translocation of cGAS to the nucleus. In the nucleus, cGAS inhibits homologous recombination of DNA double-stranded breaks, promoting tumorigenesis via a poly(ADP-ribose)-mediated interaction with PARP1 that disrupts the formation of the PARP1-Timeless complex at double-stranded break sites (Figure 2) (Liu et al., 2018). Oligomerization of dsDNA-bound nuclear cGAS into higher-ordered complexes also hinders DNA strand invasion by RAD51, an enzyme that catalyzes homologous recombination-mediated double-stranded DNA break repair (Jiang et al., 2019). Conversely, by inhibiting end-to-end fusion of short telomeres during mitosis, cGAS safeguards genomic stability and promotes replicative senescence (Li et al., 2022b). Furthermore, by binding to DNA in the nucleus, cGAS has been shown to decelerate replication forks and suppress replication-induced DNA damage (Figure 2) (Chen et al., 2020). In summary, these canonical and non-canonical pathways govern inflammation, macroautophagy, and genomic stability—processes inherent to the pathogenesis of IBD and GI malignancies.

## cGAS signaling pathways in IBD

**FIGURE 3:**
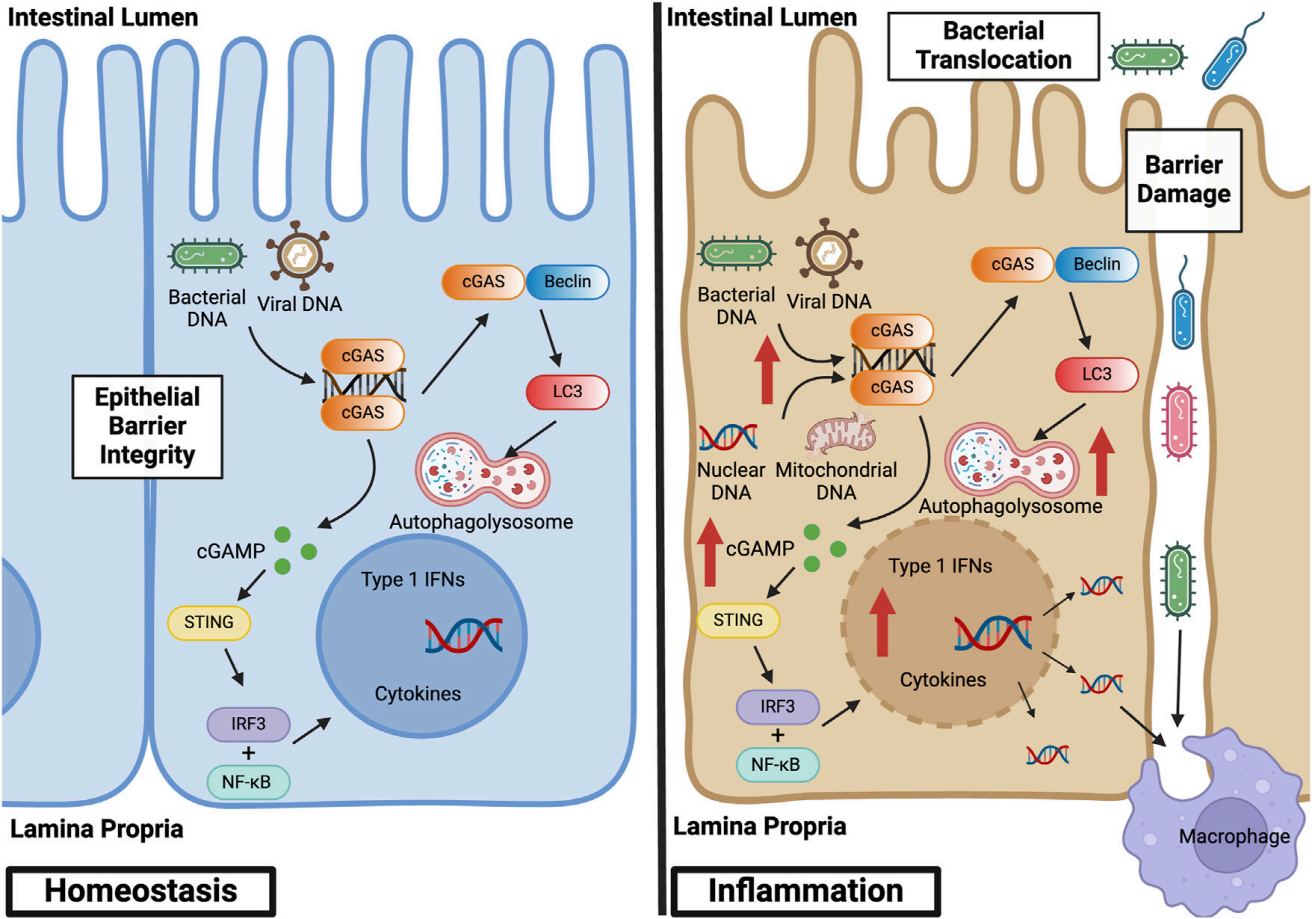
cGAS Signaling Pathways in Intestinal Epithelial Homeostasis and Inflammation. At baseline, cGAS activation within the intestinal epithelium by bacterial and viral DNA promotes defense against pathogenic infections. This activation facilitates homeostasis and the symbiotic relationship between the gut microbiome and colonic epithelium. During inflammation, however, cGAS within the intestinal epithelium is activated by both exogenous and endogenous sources of dsDNA, such as nuclear and mitochondrial DNA derived from damaged cells. This overactivation stimulates canonical and non-canonical cGAS signaling pathways, thereby inducing not only a pro-inflammatory, type 1 IFN response but also a self-regulatory, anti-inflammatory response via autophagy. Epithelial damage results in loss of barrier integrity, bacterial translocation into the lamina propria, and macrophage infiltration and activation. Created with BioRender.com.

Abnormal activation of the cGAS pathway has been implicated in the pathogenesis of a multitude of autoimmune diseases, including systemic lupus erythematosus (SLE), Aicardi-Goutières syndrome (AGS), and IBD (Liu and Pu, 2023). IBD represents a set of idiopathic, chronic, and relapsing inflammatory diseases of the GI tract. Dysregulation of normal intestinal epithelial homeostasis leads to increased intestinal permeability and exaggerated immune responses to gut microbiota driven, in part, by pattern recognition receptors, such as cGAS (Coskun, 2014). Patients with IBD exhibit increased circulating cell-free nuclear and mitochondrial DNA, as well as plasma extracellular vesicles containing dsDNA (Boyapati et al., 2018; Vrablicova et al., 2020). These endogenous sources of dsDNA, as well as exogenous sources from gut microbiota, can lead to cGAS activation. This activation subsequently influences mechanisms inherent to the pathogenesis of IBD, including abnormal immune responses, gut microbial dysbiosis, epithelial barrier dysfunction, and defects in autophagy (Rioux et al., 2007; Adolph et al., 2013; Larabi et al., 2020). Figure 3 illustrates the role of canonical and non-canonical cGAS signaling pathways in intestinal epithelial homeostasis and inflammation, and Table 1 summarizes the main findings in IBD.

**TABLE 1:** Main findings about canonical and non-canonical cGAS signaling pathways in IBD.

Main findings	Cell type/Animal model	References
**Canonical Signaling Pathway**		
*Pro-inflammatory*		
Constitutive activation of STING promotes spontaneous colitis and dysbiosis. STING accumulates primarily in colonic myeloid cells.	N153s murine model (STING gain-of-function mouse)	Shmuel-Galia et al. (2021)
WT mice subjected to DSS and subsequently treated with a STING agonist exhibit worsened colitis severity. STING-deficient mice subjected to DSS exhibit reduced colitis severity. M2 murine macrophages treated *in vitro* with a STING agonist repolarize into an M1-pro-inflammatory subtype.	WT murine model of DSS-induced colitis co-treated with DMXAA Tmem173^gt^ murine model of DSS-induced colitis (STING mutant mouse) BMDMs derived from femurs of WT mice incubated with DMXAA	Martin et al. (2019)
In models of spontaneous colitis in IL-10-KO mice, STING deficiency is associated with reduced intestinal inflammation.	IL-10^−/−^/STING^−/−^ double-deficient murine model	Ahn et al. (2017)
Inhibition of the cGAS-STING-dependent signaling pathway attenuates chemically induced colitis.	WT murine model of DSS-induced colitis co-treated with ANP or Si-Ni-San	Cai et al. (2021), Chen et al. (2021)
GSDMD deficiency in mice exacerbates chemically induced colitis. Treatment with a cGAS inhibitor ameliorates this phenotype.	GSDMD^−/−^ murine model of DSS-induced colitis co-treated with RU.521	Ma et al. (2020)
*Anti-inflammatory*		
Mice globally deficient in STING subjected to AOM and DSS are more prone to colonic inflammation and polyp formation. But mice deficient in STING only within macrophages, neutrophils, or dendritic cells and treated with AOM/DSS have less colonic inflammation and polyp formation compared to STING-global-KO mice.	STING^−/−^, LysM-STING^−/−^(deleted from macrophages and neutrophils), CD11c-STING^−/−^ (deleted from dendritic cells) murine models of AOM-DSS-induced colitis-associated colorectal cancer	Ahn et al. (2017)
STING deficiency is associated with disruption of gut homeostasis. STING KO mice demonstrate worsened intestinal inflammation when subjected to DSS-induced colitis, T-cell-induced colitis, and *Salmonella typhimurium* infection.	STING^−/−^ murine model of DSS-induced colitis, T-cell-induced colitis, and *Salmonella typhimurium* infection.	Canesso et al. (2018)
**Non-canonical Signaling Pathway**		
*Anti-inflammatory*		
When subjected to DSS, cGAS-deficient mice exhibit worsened colitis as well as downregulation of autophagy proteins.	cGAS^−/−^ murine model of DSS-induced colitis	Khan et al. (2022)
Chemically induced colitis results in destruction of intestinal stem cells and impaired intestinal barrier function in cGAS KO but not STING mutant mice.	cGAS^−/−^ murine model of DSS-induced colitis	Hu et al. (2021a)

### Canonical cGAS signaling pathway in IBD

STING expression is increased in immune and epithelial cell lineages in humans with IBD and mice with colitis (Chen et al., 2021; Shmuel-Galia et al., 2021; Flood et al., 2022). Studies examining the impact of this upregulation in IBD, however, are conflicting. Whereas multiple studies have shown that upregulation of STING exacerbates intestinal inflammation, others have reported the opposite phenomenon.

Constitutive activation of STING has been shown to promote spontaneous colitis and dysbiosis in a STING gain-of-function mouse model. Measurement of protein and transcript levels demonstrated STING to be undetectable or very low in WT control colons, but to be significantly elevated in WT mice subjected to dextran sodium sulfate (DSS) colitis. In the setting of intestinal inflammation, STING accumulates primarily in colonic myeloid cells, which is mediated by bacterial cyclic dinucleotide-induced ubiquitination of the protein (Shmuel-Galia et al., 2021). Similarly, wild-type mice subjected to DSS and subsequently treated with the STING agonist, DMXAA, exhibited worsened colitis severity as demonstrated by increased weight loss, decreased colon length, and worsened colonic damage on histology (Martin et al., 2019). Furthermore, STING-deficient mice, when subjected to DSS, have been shown to have reduced colitis severity (Chen et al., 2021). Interestingly, in mice with both STING and type I IFN receptor deficiency, the same protection from inflammation was not seen, suggesting that STING-mediated colitis may be independent of type I IFN signaling (Shmuel-Galia et al., 2021). Staining for Iba-1, a protein found to be upregulated in macrophages during activation, in sections of DSS-exposed colons was attenuated in STING deficient mice. M2 murine macrophages treated *in vitro* with a STING agonist repolarized into an M1-pro-inflammatory subtype, suggesting that STING activation may exacerbate colonic inflammation via stimulation of macrophages and a shift towards M1 polarization (Martin et al., 2019).

In models of spontaneous colitis in IL-10-knockout (KO) mice (IL-10 is a major immunosuppressive cytokine), STING deficiency again was associated with reduced intestinal inflammation, decreased production of pro-inflammatory cytokines, and a reduction in spontaneous polyp formation (Ahn et al., 2017). Similar but less robust results were found in IL-10-cGAS double-KO mice, again suggesting that cyclic dinucleotides produced directly by bacteria may play an important role in influencing STING signaling. Interestingly, however, in this same study, mice globally deficient in STING subjected to azoxymethane (AOM) and DSS were more prone to colonic inflammation and polyp formation. But mice deficient in STING only within macrophages, neutrophils, or dendritic cells and treated with AOM/DSS had less colonic inflammation and polyp formation compared to STING-global-knockout mice. These results underscore the importance of distinguishing between models utilized to study these signaling pathways, the importance of STING signaling within myeloid lineages, and the possibility of differential roles of STING within cell types (Ahn et al., 2017). The differing results may stem from STING’s dual role in which it is protective within the intestinal epithelium while pro-inflammatory in myeloid cells. Conducting analogous experiments on transgenic mice with STING specifically deleted in the intestinal epithelium or adaptive immune cells could offer additional insights into its precise function. Inhibition of the cGAS-STING-dependent signaling pathway with atrial natriuretic peptide (ANP) or the herbal supplement Si-Ni-San has also been shown to attenuate chemically-induced colitis in mice (Cai et al., 2021; Chen et al., 2021). Although the exact mechanism remains unclear, treatment with ANP resulted in decreased protein levels of cGAS and phosphorylation of STING, TBK1, and IRF3 in intestinal tissue. Increased co-localization of cGAS with NPR-A, an ANP receptor located on the plasma membrane of target cells, was demonstrated in mice treated with ANP, suggesting that ANP possibly inhibits the cGAS-STING pathway.

Gasdermin D (GSDMD), a mediator of intestinal epithelial cell pyroptosis, negatively regulates the cGAS-STING pathway in macrophages (Ahn et al., 2017). GSDMD deficiency in mice has been shown to exacerbate chemically-induced colitis. Treatment with a cGAS inhibitor ameliorates this phenotype, suggesting that GSDMD deficiency leads to increased cGAS expression and further upregulation of cGAS-STING mediated intestinal inflammation (Ma et al., 2020). In intestinal organoids, the autophagy mediator and IBD risk gene, ATG16l1, regulates IL-22 induced STING-dependent type 1 IFN signaling and promotion of epithelial cell death (Aden et al., 2018). Mice carrying an intestinal epithelial deletion of ATG16l1 that were treated with systemic IL-22 exhibited ileitis and necroptotic epithelial cell death. Blocking the type 1 IFN response with an anti-IFNAR antibody, however, ameliorated this response (Aden et al., 2018). Additionally, colonic epithelial organoids stimulated with TNFα and IFN-β or IFN-γ resulted in increased STING-induced cell death (Flood et al., 2022).

Other studies, however, have demonstrated a reduction in colitis severity with cGAS-STING activation. STING deficiency in mice has been shown to be associated with disruption of gut homeostasis as evidenced by fewer goblet cells, decreased mucous production, lower levels of secretory IgA, increased group 1 innate lymphoid cells, and a more pro-inflammatory gut microbiome. These STING KO mice demonstrated worsened intestinal inflammation when subjected to DSS-induced colitis, T-cell-induced colitis, and *Salmonella typhimurium* infection (Canesso et al., 2018). In the setting of acute intestinal injury secondary to allogenic hematopoietic stem cell transplantation, targeted activation of the STING signaling pathway via intravenous injection of IFN-stimulatory DNA promoted intestinal epithelial integrity as evidenced by reduced translocation of FITC-dextran across the gut epithelia and reduced the risk of graft-versus-host disease (Fischer et al., 2017).

The discrepancies seen between studies showing the canonical cGAS-STING pathway to induce or suppress the formation of colitis could be due to utilization of different colitis inducing models as discussed previously or differences in microbiota. Given the pronounced interaction between STING signaling and the gut flora, variations in the microbiota between facilities and mice with differing genetic backgrounds could result in inconsistencies in experimental outcomes. Furthermore, whereas some studies utilize cohousing, which allows for the transfer of microbiota between genetically differing mice, others do not.

### Non-canonical signaling pathways in IBD

Far fewer studies to date have focused on the cGAS-dependent but STING-independent signaling pathways in IBD. cGAS has been demonstrated to be upregulated in the intestinal epithelium of humans with IBD and mice subjected to DSS colitis (Khan et al., 2022). When subjected to DSS, cGAS-deficient mice showed worsened colitis and were demonstrated to have downregulation of autophagy proteins, including Beclin-1 via Western blot analysis and immunofluorescence staining. As mentioned previously, the NTase domain of cGAS has been shown to bind Beclin-1 in the presence of dsDNA, halting the production of 2′3′-cGAMP and stimulating autophagy-mediated degradation of pathogenic DNA (Liang et al., 2014). cGAS binds to Beclin-1 in intestinal epithelial cells, and loss of cGAS has been associated with decreased autophagic flux. These findings suggest that cGAS upregulates Beclin-1-mediated autophagy and thereby maintains intestinal epithelial homeostasis during human IBD and murine chemical colitis. cGAS, but not STING, is demonstrated to be highly expressed in intestinal stem cells. Chemically-induced colitis resulted in destruction of intestinal stem cells and impaired intestinal barrier function in cGAS KO but not STING mutant mice (Hu et al., 2021a). Together, these studies suggest that in addition to its pro-inflammatory effects mediated via the STING-dependent pathway, cGAS exhibits self-regulatory, anti-inflammatory properties in IBD. Further research into the mechanistic alterations of cGAS and its influence in human disease is required to elucidate this duality.

## cGAS signaling pathways in GI malignancies

GI cancers significantly impact global health due to their high morbidity and mortality rates. Each year an estimated 4.8 million people are diagnosed with a GI cancer, leading to 3.4 million cancer-related deaths annually (Arnold et al., 2020; Sung et al., 2021). As we have gained a better understanding of the etiologies of GI cancers, it has become evident that the interplay between environmental factors and biological pathways is central to their development and progression. The GI epithelium is in continuous contact with external agents, including the gut microbiota, diet, and medications—all of which can induce epithelial cell injury, DNA damage, and subsequent activation of the cGAS-STING pathway (Ke et al., 2022; Yang et al., 2023). cGAS is integral in several cellular responses to DNA damage, which can lead to the onset and progression of GI cancers or, alternatively, anti-tumor immunity (Garland et al., 2021; Samson and Ablasser, 2022). Central to these responses is the detection of free cytosolic DNA, commonly found in cancer cells (Li and Chen, 2018). Once bound to dsDNA, activated cGAS catalyzes the formation of 2′3′-cGAMP, resulting in downstream STING and IFN activation (Bai and Liu, 2019). This cascade can instigate a range of immune reactions that are essential to the identification and eradication of cancer cells (Shen et al., 2021). However, at times this stimulation can lead to paradoxical cell survival and proliferation by augmenting autophagy and fostering an immunosuppressive tumor microenvironment (Zheng et al., 2020). This dualistic nature of cGAS in GI malignancies presents many complexities but also opportunities for therapeutic targets (Khoo and Chen, 2018; Vashi and Bakhoum, 2021). Table 2 summarizes the main findings about cGAS signaling pathways in GI malignancies.

**TABLE 2:** Main findings about canonical and non-canonical cGAS signaling pathways in GI malignancies.

Main findings	Cell type/Animal model/Specimen	References
**Esophageal Cancer**		
*Pro-tumorigenic*		
TFAM deficiency was identified in ESCC tumor samples and cell lines. This lack of TFAM and its associated mitochondrial DNA leakage lead to increased cGAS mediated autophagy and promotion of ESCC growth.	ESCC patient specimens; Human ESCC cell line (KYSE-140)	Li et al. (2022c)
Drp1 overexpression in esophageal cancer cells lines leads to a subsequent increase in cytosolic mitochondrial DNA, which activates cGAS. Increased cGAS activation leads to increased autophagy and cell survival.	ESCC tissue specimens; Human ESCC cells lines (KYSE-30, KYSE-140); BALB/c nude mice injected with ESCC cells	Li et al. (2022d)
*Anti-tumorigenic*		
POLQ, a polymerase involved in double stranded DNA break repair, is found to be upregulated in ESCC. POLQ KO cells show higher levels of DNA damage, leading to hyperactivation of cGAS and upregulation of ISGs and STAT-1, suggesting a compensatory defense mechanism for innate immune system activation.	ESCC patient specimens; Human normal esophageal epithelial cell line (NE1) Human ESCC cells lines (KYSE70TS, KYSE180TS, KYSE70, KYSE180)	Li et al. (2021b)
Post-therapeutic radiation ESCC human samples show increased dsDNA and cytosolic fragments leading to activation of cGAS and its subsequent inflammatory cytokines. A significant positive correlation between increased cGAS expression and increased CD8^+^ cells after treatment suggests a possible anti-tumor environment.	ESCC patient specimens	Nakajima et al. (2023)
**Gastric Cancer**		
*Pro-tumorigenic*		
cGAS KO gastric cancer shows decreased tumor cell viability. cGAS KO mice show lower tumor burden and growth. cGAS over-expression in AGS cells leads to activation of the MRN complex, promoting genomic instability.	Human gastric cancer cell lines (AGS, MKN45); BALB/c-nude cGAS KO mice	Liu et al. (2021)
*Anti-tumorigenic*		
TCGA database analysis of gastric adenocarcinoma shows an increase in 117 cGAS related genes and ISGs. This increase highlights stimulation of the immune system against gastric cancer cells by activation of the cGAS-STING pathway.	TCGA database	Yang et al. (2021)
**Colorectal Cancer**		
*Pro-tumorigenic*		
Analysis of CRC samples shows carriers of two variants of cGAS, rs72960018 and rs9352000, and one of TMEM173, rs13153461, to have a 3-fold increased risk of CRC development.	CRC patient specimens	Catalano et al. (2020)
*Anti-tumorigenic*		
Expression levels of all cytosolic DNA sensors are decreased in colorectal tumor tissues compared to normal tissue except for cGAS. An elevation in cGAS gene expression is associated with early-stage colorectal cancers. cGAS mediated immune stimulation might contribute partially to prevent further colorectal cancer progression.	CRC patient specimens	Yang et al. (2017)
In colitis-associated cancer models, cGAS KO mice demonstrate increased intestinal inflammation, increased tumorigenesis, and tumors with higher grades of dysplasia. Colonic tumors in cGAS-deficient mice demonstrate higher ki67 and BrdU expression compared to WT mice as well as increased activation of STAT3, suggesting possible effects on tumor proliferation.	WT, cGAS KO, Tmem173^gt^, Lgr5-EGFP-IRES-creERT2, Apc^min/+^ and IFNAR1^−/−^ murine models of AOM-DSS-induced colitis-associated colorectal cancer	Hu et al. (2021a)
Upregulation of cGAS is correlated with MSI-CRC as observed in biopsies from patients with metastatic disease. Increased expression of cGAS and STING is considered an indicator of a positive immunotherapy response. Elevated levels of cGAS expression may be indicative of a better prognosis for long-term disease-free survival.	Metastatic CRC patient specimens	Kunac et al. (2022)

### Esophageal cancer

Globally, esophageal cancer is the eighth most prevalent cancer and the sixth most deadly (Then et al., 2020). In the United States alone, there are approximately 20,000 new cases annually, and it is responsible for an estimated 16,000 deaths (Siegel et al., 2019). The development of esophageal cancer is attributed to both genetic and environmental factors. Major risk factors include smoking, obesity, advanced age, alcohol consumption, and gastroesophageal reflux disease (Kamangar et al., 2009; Then et al., 2020). cGAS has been implicated in the development and progression of esophageal cancer through a variety of mechanisms (Chen et al., 2023).

Mitochondrial transcription factor A (TFAM) is a transcription factor involved in regulating mitochondrial DNA transcription, replication, and organization within the mitochondria, thereby influencing mitochondrial biogenesis and function (Kang et al., 2018). TFAM deficiency-associated dysfunction in mitochondrial biogenesis leads to increased leakage of mitochondrial DNA into the cytoplasm, which activates cGAS. A notable decrease in TFAM levels have been reported in tissue collected from patients with esophageal squamous cell carcinoma (ESCC) and the ESCC cell line, KYSE-140. This decrease disrupts mitochondrial biogenesis, a crucial process in cellular energy production and metabolic regulation. Furthermore, this allows fragmented mitochondrial DNA released into the cytosol to activate cGAS. As discussed previously, however, the influence of cGAS goes beyond DNA sensing. cGAS has also been implicated in the modulation of autophagy, a cellular degradation and recycling pathway. cGAS has been shown to promote autophagy via upregulation of LC3-II, a marker of autophagy (Li et al., 2022c; Khan et al., 2022). This relationship is further elucidated by siRNA knockdown experiments. Knockdown of cGAS has been shown to decrease LC3-II accumulation and is associated with hindered STING and p62 degradation (Li et al., 2022c). Lack of TFAM and its associated increase in cytoplasmic mitochondrial DNA led to increased cGAS-mediated autophagy and promotion of ESCC growth, shedding light on the interplay between mitochondrial dysfunction, cGAS, and autophagy in esophageal cancer (Li et al., 2022c).

Prolonged activation of the cGAS pathway has been shown to lead to immune dysfunction, as well as an immunosuppressive tumor microenvironment (Snell et al., 2017; Ng et al., 2018). Drp1, a GTPase involved in mitochondrial fission, is upregulated in esophageal cancer cells and is associated with poor overall survival (Li et al., 2022d). Drp1 overexpression in KYSE-140 cells leads to disturbed mitochondrial function and increased cytosolic mitochondrial DNA. Treating these cells with a Drp1 selective inhibitor decreases the survival of the cancer cells (Li et al., 2022d). In KYSE-30 cells, overexpression of Drp1 led to increased LC3-II protein expression and a slight decrease in SQSTM1/p62. Western blot analysis showed that the cells with increased Drp1 expression exhibited increased STING and TBK1 phosphorylation, implicating the cGAS-STING pathway in the mechanism promoting autophagy (Gui et al., 2019; Li et al., 2022d; Khan et al., 2022). To confirm this phenomenon, the authors utilized cGAS KO cells and found no increase in LC3-II, decreased degradation of SQSTM1/p62, and suppression of STING and TBK1 phosphorylation despite overexpression of Drp1 (Li et al., 2022d). Thus, the increase in cytosolic mitochondrial DNA due to Drp1 overexpression activates cGAS, leading to increased ESCC cell autophagy and survival (Li et al., 2022d).

Studies have shown that cGAS plays a dual role, contributing to both anti-tumor response and tumor escape (Barber, 2015; Li et al., 2022d). Tumor-derived DNA can lead to the activation of cGAS, resulting in cellular senescence, initiation of pro-inflammatory cascades, and IFN-1 signaling-mediated innate immune responses (Su et al., 2019; Wang et al., 2020). cGAS is crucial to the tumor immune microenvironment in ESCC (Li et al., 2021b). DNA polymerase theta (POLQ), a polymerase involved in double-stranded DNA break and replication fork repair, is upregulated in ESCC (Wang et al., 2019; Li et al., 2021b). In normal human esophageal epithelial cell line NE1, mRNA and protein levels of POLQ were both shown to be upregulated (Wang et al., 2019). When POLQ was knocked out in NE1 cells lines, significantly higher levels of DNA damage were detected in comparison to WT cell lines (Wang et al., 2019). In these cells, greater DNA damage led to hyperactivation of cGAS and its downstream signaling pathways as demonstrated by upregulation of interferon-stimulated genes (ISGs) and increased phosphorylation of STAT-1 (Wang et al., 2019). This suggests a potential connection between impaired DNA repair mechanisms and the activation of the innate immune system by the cGAS-STING-STAT1 pathway (Wang et al., 2019).

Replication stress and DNA damage caused by radiation, the mainstay of ESCC treatment, can lead to formation of micronuclei that leak into the cytoplasm (Wilhelm et al., 2019). Micronuclei, indicative of unresolved genomic instability, have also been shown to be immunostimulatory cytosolic DNA that can activate cGAS (Chen et al., 2016b; Mackenzie et al., 2017). In ESCC, radiation has been shown to augment the expression of cGAS (Nakajima et al., 2023). Radiation leads to breaks in dsDNA and an increase in cytosolic DNA fragments (Borrego-Soto et al., 2015). cGAS recognizes these radiation-induced DNA fragments and elicits the production of inflammatory cytokines and chemokines (Kwon and Bakhoum, 2020; Nakajima et al., 2023). This heightened production of inflammatory mediators leads to an influx of immune cells into the tumor microenvironment, including CD8^+^ cytotoxic T cells (Nakajima et al., 2023). In ESCC biopsies from patients before and after treatment, there is an increase in CD8^+^ T cells in the tumors after radiation (Nakajima et al., 2023). Furthermore, there was no association between cGAS and CD8^+^ T cells in ESCC tumor tissue pre-radiation, but there was a significant positive correlation between increased cGAS expression and increased CD8^+^ T cells after treatment (Nakajima et al., 2023). Interestingly, although there was a trend towards a positive correlation between STING and CD8^+^ T cells, it was not significant. These data suggest that cGAS and possibly STING play a role in the influx of CD8^+^ T cells after radiation, leading to increased anti-tumor immunity (Nakajima et al., 2023).

### Gastric cancer

Gastric cancer is the fifth most common cancer globally, as well as the third most common cause of cancer-related deaths (Cheng et al., 2016; Smyth et al., 2020). The pathogenesis of gastric cancer is multifactorial, involving a combination of genetic, environmental, and lifestyle factors (Figueiredo et al., 2017). *Helicobacter pylori* infection, which causes chronic gastritis and gastric ulcers, is a major risk factor (Wroblewski et al., 2010). Other factors, such as high-salt diet, smoking, and alcohol consumption, also play a role in the development of gastric cancer (Machlowska et al., 2020). The progression of gastric cancer is a multistep process involving the accumulation of genetic alterations and epigenetic changes that lead to the transformation of normal gastric cells into malignant cells. The molecular subtypes of gastric cancer, including intestinal and diffuse types, have distinct genetic and epigenetic profiles and respond differently to treatment (Figueiredo et al., 2017). The identification of key molecular drivers and signaling pathways involved in the pathogenesis of gastric cancer has led to the development of new targeted therapies. Due to the role of cGAS in recognizing pathogen-derived DNA and the extensive foreign pathogens and inflammatory exposures that lead to gastric cancer, studying the role of cGAS in gastric cancer has become an important area of investigation (Ke et al., 2022).

There are 117 cGAS-related genes including CXCL10, IRF3, CCL4, TLR3, and TBK1, that have been studied in patients with gastric adenocarcinoma utilizing the Tumor Cancer Genome Atlas (TCGA) to develop a predictive model for prognosis (Yang et al., 2021). Differential expression of a multitude of cGAS-STING pathway-related genes (CSRs) was discovered in cancerous tissue in patients with gastric cancer. Specifically, expression levels of ISGs, such as IFI44L and IFI44, were shown to be associated with better overall survival and disease-free survival in gastric cancer patients (Yang et al., 2021). This association hints at the possibility that the cGAS-STING pathway is involved in activation of the immune response against gastric cancer cells. Identification of CSRs could assist with prognostication or possibly prediction of response to medical therapy (Yang et al., 2021).

Similar to its dichotomous role in esophageal cancer, cGAS influences cancer immunity and progression within gastric cancer cells. Increased expression of cGAS has been found in gastric cancer tissue in comparison to normal gastric mucosa utilizing the TCGA database. There is a stepwise increase in cGAS expression during the progression from normal gastric mucosa to T4 disease (Liu et al., 2021). Using human AGS and MKN45 gastric cancer cell lines with siRNA knocked-down cGAS, it was demonstrated that cGAS-deficient gastric cells have reduced cell viability compared to non-gastric cancer cells with cGAS knocked down. Furthermore, cGAS-knockdown in MKN45 cells decreased subcutaneous tumor volume and growth rate in BALB/c-nude mice. The study then investigated the role of cGAS on the MRE11-RAD50-NBN (MRN) complex comprised of checkpoint proteins involved in DNA repair in MKN45 and AGS cells. cGAS expression and MRN complex activation were directly correlated, suggesting that cGAS could be directly involved in the formation of the MRN complex. This theory is supported by the finding that cGAS and MRE11 were detected in the immunoprecipitated lysates of AGS cell lines (Liu et al., 2021). The authors concluded that cGAS overexpression leads to the activation of the MRN complex, thereby promoting genomic instability in gastric cancer cells and leading to tumor progression. Thus, downregulation of the MRN complex leading to the deactivation of cell cycle checkpoints could be a potential target for therapy (Liu et al., 2021).

### Colorectal cancer

Colorectal cancer (CRC) is the third leading cause of cancer and the second leading cause of cancer-related deaths in the United States (Siegel et al., 2023). The development of CRC is suggested to result from complex interactions between genetic factors, inflammatory intestinal diseases, dietary factors, the gut microbiome, and environmental exposures (Rawla et al., 2019). Interestingly, although the incidence of colorectal cancer overall has remained stable, the incidence of young-onset colorectal cancer, defined as colorectal cancer in patients below the age of 50, has increased (Sifaki-Pistolla et al., 2022). One aspect of the pathogenesis of CRC is the accumulation of a variety of genetic alterations in genes, including APC, KRAS, BRAF, PIK3CA, SMAD4, and p53 (Li et al., 2021c). However, the complex interplay of these variables has sparked an avenue for studying enzymes, such as cGAS and its activation of the immune response (Ke et al., 2022).

In a case control study of 1,423 patients with CRC and 1,114 healthy controls, CGAS, TMEM173, the gene that encodes the protein STING, and TBK1, were studied. Carriers of two variants of cGAS single nucleotide polymorphisms, rs72960018 and rs9352000, and one of TMEM173, rs13153461, revealed a 3-fold increased risk of CRC (Catalano et al., 2020). Furthermore, it has been shown that epistatic interactions between multiple genes downstream of cGAS/STING are also associated with a significantly increased risk of CRC (Catalano et al., 2020). Focusing specifically on DNA-sensing and nuclease-related genes, a multitude of genes, including *RNASEH2A*, *RNASEH2B*, and *RNASEH2C*, as well as *cGAS*, *STING, TBK1*, and *IFNB1*, were studied in tumor tissue of 53 patients with CRC compared to adjacent normal tissue (Catalano et al., 2020). All cytosolic DNA-sensing and nuclease-related genes except cGAS, RNASEH2A, and RNASEH2B had decreased expression levels in colorectal tumor tissue compared to normal tissue (Yang et al., 2017). The only cytosolic DNA sensor gene found to be upregulated was cGAS. Interestingly, this elevation in cGAS gene expression was associated with early-stage colorectal cancer. No upregulation of cGAS was found in more advanced stage tumors, suggesting that although cGAS is upregulated in early-stage colon cancers, the cGAS-mediated immune stimulation might contribute partially to preventing CRC progression (Hu et al., 2021b). In a mouse model of colitis-associated colorectal cancer, AOM/DSS, which uses AOM, a carcinogen that induces DNA damage and mutations, along with DSS to establish intestinal inflammation and colonic tumorigenesis, cGAS KO mice were shown to have worsened intestinal inflammation, increased tumorigenesis, and greater dysplasia within tumors (Hu et al., 2021b; Khan et al., 2022). Intraperitoneal injection of AOM, a carcinogen that induces DNA damage and mutations, was used along with DSS to establish the experimental model of colitis-associated colorectal cancer (Hu et al., 2021b; Khan et al., 2022). This is the most used model of inflammation associated colonic tumorigenesis for murine studies. Colonic tumors in cGAS-deficient mice demonstrated higher ki67 and BrdU incorporation compared to WT mice as well as increased activation of STAT3, highlighting possible effects on tumor proliferation (Hu et al., 2021b). Furthermore, the colonic tumors in cGAS KO mice demonstrated an increase in myeloid-derived suppressive cells, Th17 differentiation, and IL-10 production (Hu et al., 2021b). Increased intestinal stem cell loss and compromised intestinal barrier function demonstrated in cGAS KO mice suggest that cGAS alters intestinal epithelial integrity, which leads to further inflammation and inflammation-associated tumorigenesis (Hu et al., 2021b). These findings suggest that cGAS provides both a protective and decelerating effect on CRC progression. When STING gt/gt and IFN-1-receptor-deficient mice were subjected to the AOM/DSS model, no significant increase in tumorigenesis was found. This finding poses the notion that cGAS impedes tumor progression in a pathway independent from the STING-IFN cascade (Hu et al., 2021b).

Metastatic CRC is associated with microsatellite instability (MSI), which often predicts a favorable response to immunotherapy (Ganesh et al., 2019). It has been observed that defective mismatch repair genes (MMR) in tumors can trigger the cGAS-STING pathway through the loss of the MutLα subunit of MLH1, leading to chromosomal instability and escape of nuclear DNA into the cytoplasm (Guan et al., 2021). Patient biopsies from metastatic CRC, categorized by either microsatellite instability or stability, show a marked elevation in cGAS and STING expression levels in the MSI group as compared to the microsatellite-stable group. This upregulation of cGAS is notably correlated with MSI CRC (Kunac et al., 2022). Analysis of both biopsy groups revealed the presence of CD4^+^ and CD8^+^ T-cells within the tumor stroma. Elevated levels of cGAS expression may be indicative of a better prognosis for long-term, disease-free survival in MSI-positive tumors (Kunac et al., 2022). Additionally, the heightened expression of cGAS and STING is considered an indicator of a positive immunotherapy response (De Roock et al., 2010; Kunac et al., 2022). Mutations in RAS family proteins, which are GTPases, are also prevalent in MSI tumors, shedding light on the potential of the cGAS-STING pathway as a therapeutic target and a biomarker for predicting the efficacy of immunotherapy in these cases (Kunac et al., 2022). cGAS can recognize genomic instability and DNA damage, key features in the onset of colon cancer. Consequently, a deficiency in cGAS could hypothetically result in unchecked cellular growth and cancer formation. The activation of the cGAS-STING pathway plays a crucial role in the surveillance and suppression of CRC progression. Nonetheless, excessive activation of this pathway can provoke a significant pro-inflammatory response, potentially resulting in detrimental outcomes.

## Therapies targeting cGAS signaling pathways

Grasping the intricacies of the cGAS signaling pathway is crucial for developing targeted therapeutics. Malfunction of the cGAS pathway has been implicated in many autoimmune disorders, inflammatory diseases, and various cancers (Zhou et al., 2023a). Manipulating the cGAS pathway offers the potential for the development of novel treatments that can selectively modulate immune responses and influence the epithelial response to various forms of damage. Efforts have led to the development of therapies that either promote or suppress cGAS activity (Hoong et al., 2020; Ou et al., 2021). cGAS inhibitors are categorized into two types: those that impede DNA binding and those that prevent the production of 2′3′-cGAMP by binding to the active site of cGAS (Hertzog and Rehwinkel, 2020; Decout et al., 2021). Conversely, cGAS activators are typically molecules that mimic DNA fragments and bind to the DNA-binding site in cGAS, leading to the production of interferons and pro-inflammatory cytokines (Motwani et al., 2019; Decout et al., 2021; Du et al., 2023). This activation bolsters the immune system’s capacity to potentiate immunogenic cell death and eliminate cancer cells (Jiang et al., 2020; Chen et al., 2022). Table 3 summarizes the various mechanisms of the therapies discussed below.

**TABLE 3:** Overview of mechanisms of action of cGAS inhibitors and stimulators.

cGAS inhibitors						
Molecule	Mechanism of action			Cell line	Mouse lineages	References
PF-06828215	Binds active site			Hybridomas of mouse splenocytes and mouse myeloma NS-1 cells		Hall et al. (2017)
RU.521	Binds catalytic site			Mouse RAW 264.7 macrophages; BMDMs from *Trex1* ^*−/−*^ mice		Vincent et al. (2017)
Anti-Malarial Drugs (AMDs)	Binds minor groove of dsDNA			Human THP-1 monocytes		An et al. (2015)
Suramin	Binds DNA-binding site			Human THP-1 monocytes		Opoku-Temeng and Sintim (2016), Wang et al. (2018a)
Suppressive Oligonucleotides - A151	Binds DNA-binding site			Human THP-1 monocytes		Steinhagen et al. (2018)

cGAS Stimulators						
Molecule	Mechanism of action			Cell line	Mouse lineages	References
Svg3	Binds DNA-binding site			Mouse RAW 264.7 macrophages; human THP-1 monocytes		Zhou et al. (2023b)
Manganese (Mn)	Unknown mechanism; co-factor for various enzymes and is involved in diverse cellular processes			Human THP-1 monocytes; human fibrosarcoma cell line (HT-1080); human PBMCs; HeLa cells	C57BL/6 Mb21d1^−/−^ (cGAS) TMEM173^−/−^ (STING) Mavs^−/−^ Irf3^−/−^Irf7^−/−^	Wang et al. (2018b)
Eribulin (ERI)	Inhibits microtubule dynamics, leading to cell cycle arrest, apoptosis in cancer cells, and ultimately increased cytosolic DNA			Human breast cancer cell line (MM231); immortalized human retinal pigment epithelial cells (RPE1)		Yamada et al. (2023)

### cGAS inhibitors

Erroneous activation of cGAS has been implicated in a multitude of autoimmune diseases, including systemic lupus erythematous and Aicardi-Goutières Syndrome, positioning cGAS inhibition as a potentially significant therapeutic strategy (Chu et al., 2021). To identify the direct inhibitors of cGAS, nuclear magnetic resonance (NMR) screening was performed leading to the discovery of PF-06928215. This compound has been shown to have the best binding affinity by surface plasmon resonance (SPR) at 0.2 μm to the cGAS active site, increased inhibitory potency in a cGAS assay with an IC50 value of 4.9 μm, and the tightest binding capability by NMR (Hall et al., 2017). However, PF-06928215 did not exhibit inhibition in cellular cGAS assays using hybridomas of isolated mouse spleen cells and mouse myeloma NS-1 cells (Hall et al., 2017). Modifying several properties, such as plasma permeability and protein-binding profiles, could potentially improve the efficacy of PF-06928215, eventually leading to alterations in downstream interferon signaling (Hall et al., 2017).

In the search for enzymatic inhibitors of cGAS, a category of compounds that attach to the catalytic pocket of cGAS was identified (Vincent et al., 2017). Within this group, RU.521 has been shown to have the highest potency and selectivity for cGAS (Vincent et al., 2017). Cellular assays using mouse RAW 264.7 macrophages demonstrated that RU.521 can inhibit cGAS-mediated interferon activation but has minimal impact on cGAS-independent pathways. Therefore, inhibitory effects of RU.521 are mediated by its selective inhibition of cGAS rather than broad innate immune modulators. In mouse models mimicking human Aicardi-Goutières Syndrome, RU.521 was shown to lead to decreased IFN gene expression levels in bone marrow-derived macrophages (Vincent et al., 2017). However, although the dsDNA-sensing mechanism of cGAS is conserved between mice and humans, mouse and human cGAS share only 60% of amino acid identity (Lama et al., 2019). With this knowledge, a high-throughput screen was utilized to identify inhibitors of human-cGAS. This high-throughput screen was performed by utilizing a chemiluminescence assay that measures ATP consumption (Lama et al., 2019). A library of 300,000 compounds was screened, and a series of compounds known as G compounds were found to be promising human-cGAS inhibitors in primary human macrophages. These compounds could potentially be instrumental in advancing our knowledge and treatment of cGAS in human disease (Lama et al., 2019).

Apart from developing novel therapeutics, research on repurposing existing drugs has shed light on alternative methods to modulate the cGAS pathway. Anti-malarial drugs (AMDs) have been utilized for decades to treat not only malaria, but also various autoimmune diseases, such as rheumatoid arthritis, Sjögren’s syndrome, and systemic lupus erythematosus (SLE) (Gurova, 2009; Ehsanian et al., 2011). A computational analysis through *in silico* screening of drug libraries discovered several AMDs that interact with the cGAS/dsDNA complex and effectively inhibit IFN production. AMDs, such as quinolone, can intercalate and bind to the minor groove of dsDNA in a way that obstructs cGAS binding (An et al., 2015). Using an electrophoretic mobility shift assay (EMSA), it has been demonstrated that pre-incubating cGAS with DNA and then introducing AMDs can disrupt the cGAS-DNA interaction in a dose-dependent manner. To assess the physiologic relevance of AMDs, THP-1 cells were transfected with dsDNA and then treated with AMDs. The results demonstrated a reduction in IFN-β production following the treatment with AMDs (An et al., 2015). Despite theoretically showing promise, the non-specific binding of these compounds to nucleic acids increases the probability of unintended interactions and potential side effects.

Suramin, a medication already employed for diseases such as river blindness and African sleeping sickness, has shown efficacy in inhibiting the cGAS pathway (Opoku-Temeng and Sintim, 2016). A screening of a small library of compounds, selected based on structural similarity to ATP and potential to inhibit dsDNA, was conducted (Wang et al., 2018a). An initial screening threshold of 30% enzymatic inhibition revealed suramin. As a polyanion, suramin does not bind strongly to DNA, leading to the hypothesis that it attaches to the DNA-binding site rather than the DNA itself (Wang et al., 2018a). An EMSA performed with labelled DNA demonstrated that increasing concentrations of suramin resulted in increasing DNA band intensity, indicating reduced cGAS binding. Suramin was also able to disrupt binding even when cGAS and DNA were pre-incubated. In THP-1 cells stimulated with dsDNA and then treated with suramin, IFN-β mRNA expression was decreased compared to controls (Wang et al., 2018a). With a dose of five uM of suramin, IFN-β expression levels mimicked those of unstimulated cells. THP-1 cells deficient in cGAS treated with either dsDNA or 2′3′-cGAMP followed by suramin exhibited no significant reduction in IFN expression, underscoring the selective inhibition of cGAS over STING or downstream modulators (Wang et al., 2018b).

Suppressive oligonucleotides (ODNs) with repetitive sequences similar to those found in mammalian telomeres have been shown to have immunosuppressive properties (Steinhagen et al., 2018). These ODNs can mimic the structure of telomeric DNA and inhibit a multitude of inflammatory conditions (Bayik et al., 2016). Synthetic ODN, A151, which was previously shown to bind to unmethylated CpG DNA and block TLR-9 activation (Gursel et al., 2003), has now also been recognized as a competitive inhibitor of cGAS, leading to downstream decreased interferon production. Both cGAS KO THP-1 and WT THP-1 cells were pretreated with A151 or a control ODN (c151) and then transfected with dsDNA, mitochondrial DNA, or 2′3′-cGAMP. Pre-treatment with A151 resulted in a substantial decrease in IFN-β mRNA levels in cells treated with dsDNA and mitochondrial DNA, but not in those cells treated with 2′3′-cGAMP. Additionally, protein analysis indicated diminished p-IRF production in cells treated with dsDNA and mitochondrial DNA but not 2′3′-cGAMP, suggesting that A151 exerts its inhibitory effects on the cGAS pathway at a stage upstream of STING (Steinhagen et al., 2018).

### cGAS stimulators

Whereas elevated levels of cGAS have been associated with a variety of autoimmune diseases, such as SLE, AGS, and IBD, lack of cGAS has been linked with several types of cancer (Hu et al., 2021b; Skopelja-Gardner et al., 2022). Immunotherapy that modifies checkpoint blockades has improved treatment outcomes for a growing number of cancers, yet a significant number of cancer patients have not benefitted from these treatments (Sanmamed and Chen, 2018). The application of immunostimulants holds considerable promise in addressing these challenges. Svg3, a cGAS-specific ODN agonistic, has been found to activate cGAS and may prove useful as an anti-cancer immunotherapy (Zhou et al., 2023b). dsDNA that is 45 base pairs in length or longer can activate cGAS by forming enzymatically active dimers; however, even shorter dsDNA with guanosine-rich overhangs can also trigger cGAS. Svg3 is a potent cGAS agonist, albeit one that consists of single-stranded DNA (ssDNA) with a hairpin structure. RNA sequencing has shown that Svg3 induces a series of ISGs without provoking any unrelated inflammasomal responses in RAW 264.7 macrophages (Zhou et al., 2023b). cGAS-deficient RAW264.7 macrophages pre-treated with Svg3 continued to exhibit a lack of interferon response compared to WT macrophages, indicating a reliance on cGAS for interferon activation (Zhou et al., 2023b). In human THP-1 monocytes and cells derived from head and neck squamous cell carcinoma, transfection with Svg3 resulted in an upregulation in ISGs, indicating (Zhou et al., 2023b) the clinical potential of Svg3 (Zhou et al., 2023b).

Transition metals are essential for all forms of life, and an estimated 30% of enzymes require them as cofactors (Ackland et al., 2015; Nriagu et al., 2015; Radin et al., 2016; Wang et al., 2018b). Manganese (Mn) plays a pivotal role in the cGAS pathway in a dsDNA-dependent manner (Wang et al., 2018b). Mn treatment in cells or mice has shown marked resistance to viral infections, a phenomenon linked to the activation of cGAS. Treatment of HeLa cells or WT mice with Mn induced IRF phosphorylation, IFN-1 production, and an upregulation of ISGs. However, this induction did not occur in the absence of cGAS and STING (Wang et al., 2018b). This effect was unique to Mn, as it significantly triggered interferon responses and boosted 2′3′-cGAMP production even at low concentrations, unlike other metals like magnesium (Wang et al., 2018b). Additionally, Mn has been observed to enhance the infiltration of CD8^+^ and CD4^+^ T cells into tumors, underscoring its essential role in maintaining the host’s anti-tumor immune response. In cGAS- and STING-deficient mice, Mn treatment did not lead to an increase in CD8^+^ or CD4^+^ T cells, thus demonstrating Mn’s reliance on the cGAS pathway to alter the tumor microenvironment (Wang et al., 2018b).

Eribulin (ERI), a microtubule polymerization inhibitor, is employed in the treatment of metastatic breast cancer (Cortes et al., 2011). Treatment of the human breast cancer cell line, MM231, and immortalized retinal pigment epithelial cell line, RPE1, with ERI resulted in heightened expression of cGAS, STING, p-IRF3, and IFN-β genes (Yamada et al., 2023). Moreover, ERI-treated cells exhibited increased cytoplasmic and nuclear localization of cGAS. These results indicate that cells treated with ERI show an increased activation of the cGAS-STING pathway compared to those treated with a control (Yamada et al., 2023).

Nanocomposites are an emerging and promising tool used for targeted delivery of immunotherapy. With the ability to interact with the immune system, they aim to overcome the current limitations of immunotherapy, such as immune evasion and systemic toxicity (Chen et al., 2022; Sharma and Otto, 2024). The application of nanocomposites to simultaneously target mitochondrial and nuclear DNA results in an increased cGAS-mediated upregulation of innate immunity and an influx of tumor-infiltrating lymphocytes in gastric cancer. The targeted activation of cGAS and subsequent enhancement of anti-tumor immunity by nanocomposites highlight their substantial potential in cancer treatment (Guo et al., 2022).

In another investigation of immunotherapy pertaining to cGAS and gastric cancer, it was found that anlotinib treatment, a tyrosine-kinase inhibitor used in various solid tumors, leads to downregulation of programmed cell death ligand (PD-L1) and activation of the cGAS/STING pathway in gastric cancer cells (Yuan et al., 2022). PD-L1, a transmembrane protein, plays a role in modulating cytokine secretion and inducing apoptosis within the immune system (Han et al., 2020). Recent studies indicate that cGAS-STING pathway activation can promote PD-L1 expression on cancer cells, potentially providing a means for these cells to elude the anti-tumor response (Kwon and Bakhoum, 2020; Lu et al., 2023). Western blot analysis of HS746T gastric cancer cells treated with anlotinib exhibited reduced PD-L1 expression when compared to untreated cells. Additionally, there was a notable increase in the expression of cGAS, STING, and IFN-β in the anlotinib-treated group relative to the control. These results point to a potential mechanism where Anlotinib treatment impedes gastric cancer development by decreasing PD-L1 expression through the cGAS/STING pathway (Yuan et al., 2022).

In summary, advancements in cGAS stimulators, such as Svg3, manganese, and eribulin, as well as the emergence of nanocomposites for targeted drug delivery have proven promising for enhancing immune responses against diseases, particularly cancers, by effectively targeting the cGAS-STING pathway.

## Discussion

In 2013, the Chen lab first described the NTase cGAS and its endogenous second messenger, 2′3′-cGAMP (Sun et al., 2013; Wu et al., 2013). Since then, a growing body of research has emerged detailing cGAS’s canonical and, more recently, non-canonical signaling pathways. Over time, it has become evident that cGAS can not only trigger an innate immune response via activation of STING, but also dampen innate immune surveillance via Beclin-1-mediated macroautophagy. Similarly, cGAS has been shown to promote tumorigenesis by inhibiting homologous recombination of double-stranded DNA breaks, safeguard genomic stability by decelerating replication forks, and suppress replication-induced DNA damage. These dual functions stress cGAS’s pivotal role in homeostasis and inflammation, and disequilibrium between these states contributes to the pathogenesis of disease processes, such as IBD and GI malignancies. This review highlights the significant overlap in the role of cGAS signaling pathways in GI malignancies and IBD. Both conditions demonstrate how altered cGAS activity is intricately linked to chronic inflammation and immune modulation. Activation of cGAS in response to cytosolic DNA leads to downstream effects via the canonical STING pathway and non-canonical signaling molecules critical in both exacerbation of inflammation in IBD and enhancement of the tumor microenvironment in cancers. Therapeutically, this suggests the potential cross-applicability of cGAS inhibitors, which could dampen hyperactive immune responses in IBD or modify the tumor microenvironment in GI cancers, presenting a dual benefit. Targeting cGAS presents a promising avenue for cancer therapy; however, there are inherent risks to these therapies. Systemic inhibition of cGAS could potentially suppress necessary immune surveillance mechanisms, leading to increased susceptibility to infections or the progression of other malignancies. The balance between therapeutic benefits and potential adverse effects must be carefully balanced. The dual role of cGAS in promoting and suppressing tumorigenesis, depending on the cellular context and disease state, further complicates the potential outcomes of targeting this pathway. Limitations to the current literature are such that evidence for cGAS stimulators predominantly arises from studies utilizing cancer or macrophage cell lines. Bridging the gap between these preclinical findings and clinical applicability is essential. While cell line studies provide valuable insights into molecular mechanisms, they often do not capture the full systemic interactions and potential side effects of cGAS modulation in a whole organism. Future studies should aim to evaluate the efficacy and safety of cGAS stimulators in animal models, subsequently assessing their therapeutic potential and limitations in human clinical trials. Lastly, consideration of the genetic and functional differences between human and mouse cGAS is crucial. Due to there being only 60% amino acid identity shared between human and mouse cGAS, all of these data necessitate cautious interpretation as mouse models may not fully translate to human biology. This underlines the importance of developing human-specific cGAS assays and considering these species differences in the early stages of drug development.
